# Reasons behind sickness presenteeism: insights from four national surveys in Latvia

**DOI:** 10.3389/fpubh.2025.1549076

**Published:** 2025-03-07

**Authors:** Svetlana Lakiša, Inese Gobina, Ivars Vanadziņš, Linda Matisāne

**Affiliations:** ^1^Institute for Occupational Safety and Environmental Health, Riga Stradinš University, Riga, Latvia; ^2^Department of Public Health and Epidemiology, Riga Stradinš University, Riga, Latvia; ^3^Department of Occupational and Environmental Medicine, Riga Stradinš University, Riga, Latvia

**Keywords:** sickness presenteeism, sickness behavior, workplace replacement challenges, drivers of presenteeism, self-reported presenteeism reasons

## Abstract

**Introduction:**

Sickness presenteeism—working despite being ill—is influenced by work demands, personal circumstances, and socio-demographic factors. This study investigates the prevalence of sickness presenteeism and its self-reported reasons across socio-demographic groups in Latvia.

**Methods:**

Data were pooled from four consecutive cross-sectional surveys conducted from 2006 to 2018, representing a sample of 6,368 hired workers. Logistic regression was used to assess associations between presenteeism and socio-demographic variables (gender, age, education, income, and job position), while chi-squared tests examined differences in reasons for presenteeism.

**Results:**

Overall, 11% of respondents reported working while ill in the past year. The most common reasons were a lack of replacement possibilities (27.7%), financial considerations (25.5%), specific work-related aspects (17.4%), and mild illness (14.0%). Significant socio-demographic differences were observed, with gender, income level, and job position influencing the reasons for presenteeism.

**Discussion:**

These findings underscore the need for targeted workplace policies, including strategies to address replacement gaps, financial insecurity, and job-related pressures. Future research should explore cross-national comparisons and the impact of evolving work patterns, such as telework, on presenteeism trends.

## 1 Introduction

Sickness presenteeism, the act of working while ill, is a growing concern due to its significant implications for employee wellbeing and organizational performance. Sickness presenteeism can be defined as continuing to attend work during illness (1). According to the report of the sixth European Working Conditions Survey (EWCS), the prevalence of sickness presenteeism in Latvia in 2015 was around 33%. In general, significant differences have been identified between countries in terms of reported sickness presenteeism (between 20 and 70%). In most countries, the association between low levels of sickness absenteeism and high levels of presenteeism has also been found (2).

Workers facing illness must decide between attending work while unwell (sickness presenteeism) or taking a leave of absence (sickness absenteeism) (1). The decision to work while ill is shaped by both health-related and non-health-related factors (3). Non-health-related factors, such as organizational and psychosocial influences, alongside an individual's socio-demographic background, play a crucial role in the decision to work while ill (4, 5).

Research indicates that women are more likely to report both working while ill and having sickness absence (2, 6–9). Differences in health-related behaviors and traditional gender roles are frequently cited as key explanations for this disparity.

Older adults are more likely to experience chronic conditions such as diabetes, hypertension, cardiovascular diseases, obesity, and respiratory illnesses, increasing their vulnerability to other health issues. Studies suggest that younger workers are more likely to engage in sickness presenteeism compared to older workers, even after adjusting for health and work-related variables (3, 5–7, 10) and work-related variables (11). The sixth European Working Conditions Survey (EWCS) reported that workers aged 50 and above were less likely to engage in presenteeism (39%) compared to workers under 35 (44%). Younger workers often prioritize entrepreneurial goals and work-life balance more than older employees (12).

The impact of educational level on presenteeism prevalence remains inconsistent across studies. Some studies report higher rates of presenteeism among individuals with higher education (7, 13), potentially due to reduced acceptance of sick leave in senior roles or limited replacement options for specialized positions (14, 15). Other studies, however, find no consistent patterns linking education level to presenteeism (16). Low income increases the likelihood of sickness presenteeism, often driven by concerns about income loss during sickness absence (17). This group also tends to experience higher job insecurity, particularly during periods of elevated unemployment (17). Economic hardships are strongly associated with higher rates of sickness presenteeism (16, 18, 19).

Research indicates that sickness presenteeism is less common among those new to a job but tends to increase with years of work experience. This trend may reflect increasing loyalty to the employer and identification with the company or growing difficulties in the possibility of replacement (14). However, lower presenteeism at the beginning of an employment relationship does not correspond to higher effort (in the form of sickness presenteeism) during the first year in a job, as workers have to gain a reputation among colleagues and management to keep contracts permanent (20).

An analysis of job position and presenteeism in 35 European countries found higher rates of presenteeism among individuals in senior positions (2). Managers and highly educated individuals may report higher presenteeism due to a perceived sense of indispensability or a desire to set an example for their subordinates (21), and they aim to function as role models for their subordinates (14). Such behaviors may also stem from the expectation to maintain productivity and uphold organizational standards. However, some studies report no significant differences in presenteeism rates among white-collar, pink-collar, and blue-collar workers (6) or across job positions such as managerial, routine non-manual, and skilled roles (5). These inconsistencies suggest that presenteeism may be influenced by other factors, such as workplace culture or sector-specific demands, beyond job position alone. For example, presenteeism is more prevalent in jobs where attendance has a great influence on other people, such as in the education or healthcare sectors (10), as well as senior positions often have higher workloads and deadlines, which are frequently mentioned as reasons for being sick at work (22). These findings highlight the importance of role-specific pressures in shaping presenteeism behavior, particularly in service-oriented professions. Consequently, research findings on employment-related risk factors for sickness presenteeism remain inconsistent. This variability underscores the need for further investigation into how job-specific demands and organizational policies influence presenteeism.

Over the past two decades, sickness presenteeism has been a prominent research focus, yet studies report varying results on its association with socio-demographic factors, often shaped by country-specific influences such as economic development, labor laws, and workplace culture (3, 11, 15, 23). Further research is required to refine presenteeism measurement methods and provide more precise insights into the factors influencing this behavior. Such research should also explore how socio-demographic and organizational factors interact to shape presenteeism across different cultural and economic contexts. This study aims to address these gaps by examining how socio-demographic factors influence presenteeism within the context of the Latvian labor market.

## 2 Materials and methods

### 2.1 Description of the study population and the sample

This study employed a cross-sectional design to examine the relationship between socio-demographic factors and self-reported sickness presenteeism. Data were pooled from four consecutive nationwide surveys on *Work Conditions and Risks in Latvia*, which were conducted in 2006 (24), 2010 (25), 2013 (26), and 2018 (27). The primary objective of these surveys was to evaluate changes in Latvia's occupational safety and health systems over time.

Respondents were randomly selected to ensure a representative sample across all regions and occupational sectors in Latvia. Only employees (hired or paid workers) were selected as the study population for this research and included in the data analysis; self-employed workers, workers on maternity leave, etc., were excluded from our analysis. Table 1 provides a detailed description of the study sample.

**Table 1:** Description of the study population.

**Year**	**Number**, ***n*** **(%)**		
	**Respondents included in analyses**	**Sickness presentees**	**Respondents in the reference group**
2006	1,601 (63.5)	166 (10.4)	1,435 (89.6)
2010	1,578 (63.0)	218 (13.8)	1,360 (86.2)
2013	1,579 (61.7)	192 (12.2)	1,387 (87.8)
2018	1,610 (64.4)	122 (7.6)	1,488 (92.4)
Total	6,368 (63.1)	698 (11.0)	5,670 (89.0)

In total, 6,368 respondents were included in the analysis. The mean age was 42.9 (SD +/– 12.6), aged between 16 and 80. 47.1% of the respondents included in the analyses were males, and 52.9%—females.

Data were collected using Computer-Assisted Personal Interviews (CAPI), with identical questions used across surveys to measure socio-demographic factors and sickness presenteeism. The standardized methodology across all surveys allowed consistent comparisons over the study period. Although the surveys spanned 12 years, the consistent methodology ensures comparability, further since the data were adjusted by survey year in the analysis; however, results may not fully represent present-day conditions.

### 2.2 Study variables

Self-reported sickness presenteeism (referred to as presenteeism hereinafter) within the previous year is the outcome variable of this study. It was measured by a single question addressed to all respondents: “Which of the following situations regarding ill-health within the previous year apply to you personally?” The answers included the following options: “I was ill and took medically certified sickness absence;” “I was ill, but did not take medically certified sickness absence;” “I was ill, but I went to work (worked) while being ill” (sickness presentees), “I was not ill within the previous year.” Two groups of respondents were included in this analysis—(1) sickness presentees and (2) those reporting not being ill within the previous year (reference group).

For studying the reasons for sickness presenteeism, an open-ended question was used: “Why did you go to work/work while being ill?” This question was asked only to those respondents who responded that they have had such experience within the previous year. On average, a total of 9.9% (*n* = 628) of respondents reported the reasons for sickness presenteeism.

The open-ended answers on sickness presenteeism reasons were coded by researchers and occupational health and safety practitioners into the following groups:

1) no possibilities for replacement (examples of quotes—“We had a lot to do and others were also ill, so I decided not to have a sick leave,” “Who will work instead of me?”);
2) financial considerations (e.g., lower income during sickness absence, no bonus payment, salary depending on the amount of work done—if during sickness absence no work is done, only minimum salary is paid etc. (“Because of money, I had a lot of bills,” “My employer does not pay sick leave”);
3) specific aspects of work (e.g., too much work, urgent work, very specific work etc. [“There was a lot to do, and nobody can replace me (because of my specific skills),” “I did not want to ask my colleagues to make a presentation as I had to present myself”];
4) sense of responsibility, or feeling guilt (“I did not want to put pressure on colleagues,” “I am a responsible person, I like to finish my tasks”);
5) fear of losing a job [e.g., firing in case of illness (“I can be fired,” “Bosses do not like workers who take sick leaves”)];
6) the worker did not want to visit a doctor (“I don't like going to doctors,” “I am too lazy to go to the doctor”); and
7) mild illness (“It was just a small cold, it did not disturb me,” “I broke my leg, so I worked from home,” “I was not so seriously ill to stay at home”).

Initially, the codes were suggested by the company responsible for organizing worker interviews for the first survey of *Work Conditions and Risks in Latvia* and confirmed by researchers and occupational health and safety practitioners. For the next surveys, the practitioners reviewed the answers to see if there were no major changes in the answers. As such changes were not identified, the same coding principles were applied for all four survey periods.

The following sociodemographic variables were studied: gender, age, education, salary, job position and work experience with current employer. Six age groups were used: 18–24, 25–34, 35–44, 45–54, 55–63, and 64–80. Preschool or incomplete primary, primary, secondary, vocational secondary, or higher educational levels were analyzed. The following job position categories were also studied: head of the company, senior/middle manager, senior and intermediate level specialist, service and sales worker, skilled worker, and unskilled worker. The question regarding the work experience with the current employer originally had the following answers: <1 month, <6 months, 6 months to 1 year, 1 to 2 years, 2 to 5 years, 5 to 10 years, and more than 10 years. Re-grouping these answers was done in the following categories: <1 year, 1 to 5 years, 5 to 10 years, 10 years and more.

Income quartiles (based on monthly salary reports) in each respective survey year were used to study the association between sickness presenteeism and income groups.

### 2.3 Statistical analysis

Data from all four surveys were combined into a single dataset for further analysis. Descriptive frequency parameters were calculated using chi-squared tests to describe and compare the data. Binomial logistic regression was performed to examine the association between presenteeism and socio-demographic parameters. The results were presented as odds ratios (ORs) and adjusted odds ratios (aORs) with 95% confidence intervals (CIs), adjusted for gender, age, education, and the survey year.

The multicollinearity of independent variables was assessed using the Spearman correlation coefficient, revealing no significant multicollinearity. Data analysis was conducted using IBM SPSS Statistics version 27 (IBM Corporation, Armonk, New York, USA). The multicollinearity between the independent variables was tested. Spearman correlation coefficient was calculated, and no significant multicollinearity was found. The IBM SPSS Statistics 27 (IBM Corporation, Armonk, New York, NY, USA) software was used for the data analysis.

## 3 Results

A total of 11.0% (*n* = 698) of respondents reported sickness presenteeism, with prevalence rates varying between 7.6 and 13.8% across the four survey years (2006–2018). Table 2 presents the prevalence and adjusted odds ratios (aORs) of sickness presenteeism across different socio-demographic groups. Significant associations were found for gender, age, education, income, and work experience.

**Table 2:** The odds of sickness presenteeism within the previous year in association with socio-demographic factors.

	**Distribution of the socio-demographic factors, % (*n*) 95% CI**	**Sickness presenteeism, OR (CI 95%)^a^, Unadjusted**	**Sickness presenteeism, aOR (CI 95%)^a^, Adjusted**
**Gender**			
Female	52.9 (3,369) 51.7–54.1	1.36^***^ (1.16–1.59)	1.34^***^ (1.14–1.58)
Male	47.1 (2,999) 45.9–48.3	1	1
**Age**			
64–80 years	3.8 (242) 3.4–4.3	0.64 (0.34–1.18)	0.59 (0.32–1.09)
55–63 years	17.4 (1,105) 16.4–18.3	1.25 (0.88–1.76)	1.22 (0.86–1.74)
45–54 years	25.9 (1,648) 24.8–27.0	1.12 (0.80–1.56)	1.05 (0.75–1.48)
35–44 years	24.1 (1,537) 23.1–25.2	1.48^*^ (1.06–2.06)	1.45^*^ (1.04–2.02)
25–34 years	20.1 (1,278) 19.1–21.1	1.53^*^ (1.09–2.14)	1.51^*^ (1.08–2.13)
18–24 years	8.8 (558) 8.1–9.5	1	1
**Education**			
Preschool or incomplete primary education	0.5 (34) 0.4–0.7	0.91 (0.32–2.60)	1.24 (0.43–3.58)
Primary education	7.7 (489) 7.1–8.4	0.81 (0.59–1.12)	0.87 (0.64–1.23)
Secondary education	21.3 (1,358) 20.3–22.4	0.86 (0.69–1.07)	0.89 (0.72–1.11)
Vocational secondary education	40.2 (2,563) 39.1–41.5	0.72^*^ (0.60–0.87)	0.74^**^ (0.61–0.90)
Higher education	30.2 (1,924) 29.1–31.3	1	1
**Salary**			
1st quartile (lowest)	25.1 (1,464) 24.0–26.3	0.68^***^ (0.54–0.86)	0.75^*^ (0.58–0.98)
2nd quartile	21.9 (1,276) 20.9–23.0	0.79^*^ (0.63–0.99)	0.81 (0.63–1.04)
3rd quartile	29.0 (1,690) 27.9–30.2	0.71^**^ (0.57–0.89)	0.75^*^ (0.60–0.95)
4th quartile (highest)	23.9 (1,393) 22.8–25.0	1	1
**Job position**			
Head of the company, senior or middle manager	8.5 (540) 7.8–9.2	1.05 (0.78–1.40)	1.09 (0.81–1.47)
Senior and intermediate level specialist	31.2 (1,978) 30.0–32.3	1	1
Service and sales employee	17.5 (1,111) 16.6–18.5	0.99 (0.79–1.24)	1.00 (0.78–1.29)
Skilled worker	30.1 (1,913) 29.0–31.3	0.80^*^ (0.65–0.97)	0.95 (0.74–1.22)
Unskilled worker	12.7 (804) 11.9–13.5	0.76^*^ (0.58–0.99)	0.84 (0.61–1.15)
**Work experience with current employer**			
<1 year	17.3 (1,092) 16.4–18.3	1	1
1 to 5 years	34.6 (2,178) 33.4–35.8	1.41^*^ (1.09–1.81)	1.35^*^ (1.04–1.75)
5 to 10 years	19.7 (1,243) 18.8–20.7	1.46^**^ (1.11–1.92)	1.37^*^ (1.03–1.84)
10 years and more	28.3 (1,784) 27.2–29.5	1.48^**^ (1.14–1.92)	1.46^*^ (1.09–1.94)

^a^The reference category for the sickness presenteeism group is a group of respondents, who did not get sick in the previous year.

aOR, odds ratios adjusted for gender, age, education, and survey year. ^*^p<*0.05*, ^**^p<*0.01*, ^***^p<*0.001*.

The odds of sickness presenteeism were higher for females (aOR = 1.34). Higher odds of presenteeism have been identified among respondents belonging to age groups of 25–34 years (aOR = 1.51) and 35–44 years (aOR = 1.45) in comparison with those aged between 18 and 24. Respondents with vocational secondary education had the lowest odds of presenteeism (aOR = 0.74), while no significant differences were observed in other educational groups compared to those with higher education. The odds for presenteeism were higher among respondents in the highest income quartile, compared to other income levels. Unskilled and skilled workers (OR = 0.76, and OR = 0.80) had lower odds of presenteeism if compared to the job position “Senior and intermediate level specialist,” but no significant difference was found after adjustment for gender, age, education, and survey year. The odds of presenteeism increased with working experience—years worked for the current employer.

On average, one-fourth of sickness presentees have reported a lack of possibilities for replacement (27.7%) and financial considerations (25.5%). 17.4% of respondents have mentioned different work-specific aspects, and an additional 14.0%—that illness was mild. Other reasons were highlighted by <10% of respondents. For details see Table 3.

**Table 3:** The distribution of the reasons reported by workers who were working ill in relation to sociodemographic factors.

	**Distribution, % (*n*)**	**No possibilities for replacement, % (*n*)**	**Financial considerations, % (*n*)**	**Specific aspects of work, % (*n*)**	**Sense of responsibility or feeling guilty, % (*n*)**	**Fear of losing job, % (*n*)**	**Did not want to visit the doctor, % (*n*)**	**Mild illness, % (*n*)**
**Total**	628	27.7 (174)	25.5 (160)	17.4 (109)	5.9 (37)	7.0 (44)	2.5 (16)	14.0 (88)
**Gender**								
Female	59.1 (371)	29.4 (109)	22.4 (83)	18.1 (67)	5.4 (20)	8.1 (30)	2.7 (10)	14.0 (52)
Male	40.9 (257)	25.3 (65)	30.0 (77)	16.3 (42)	6.6 (17)	5.4 (14)	2.3 (6)	14.0 (36)
**Age** ^**^								
64–80 years	1.8 (11)	0.0 (0)	9.1 (1)	18.2 (2)	27.3 (3)	27.3 (3)	18.2 (2)	0.0 (0)
55–63 years	16.7 (105)	28.6 (30)	17.1 (18)	24.8 (26)	4.8 (5)	10.5 (11)	2.9 (3)	11.4 (12)
45–54 years	23.9 (150)	29.3 (44)	30.7 (46)	16.0 (24)	2.7 (4)	3.3 (5)	4.7 (7)	13.3 (20)
35–44 years	28.3 (178)	30.3 (54)	26.4 (47)	15.7 (28)	7.3 (13)	6.2 (11)	1.1 (2)	12.9 (23)
25–34 years	22.5 (141)	27.0 (38)	27.0 (38)	15.6 (22)	6.4 (9)	7.1 (10)	1.4 (2)	15.6 (22)
18–24 years	6.8 (43)	18.6 (8)	23.3 (10)	16.3 (7)	7.0 (3)	9.3 (4)	0.0 (0)	25.6 (11)
**Education** ^**^								
Preschool or incomplete primary	0.6 (4)	25.0 (1)	25.0 (1)	0.0 (0)	0.0 (0)	25.0 (1)	0.0 (0)	25.0 (1)
Primary	7.3 (46)	23.9 (11)	39.1 (18)	10.9 (5)	0.0 (0)	8.7 (4)	4.3 (2)	13.0 (6)
Secondary	22.3 (140)	33.6 (47)	26.4 (37)	11.4 (16)	4.3 (6)	10.0 (14)	0.7 (1)	13.6 (19)
Vocational secondary	35.2 (221)	24.0 (53)	28.5 (63)	15.4 (34)	5.0 (11)	8.6 (19)	4.1 (9)	14.5 (32)
Higher	34.6 (217)	28.6 (62)	18.9 (41)	24.9 (54)	9.2 (20)	2.8 (6)	1.8 (4)	13.8 (30)
**Salary** ^***^								
1st quartile (lowest)	21.5 (126)	30.2 (38)	22.2 (28)	15.9 (20)	6.3 (8)	11.9 (15)	3.2 (4)	10.3 (13)
2nd quartile	22.4 (131)	38.9 (51)	29.8 (39)	6.1 (8)	1.5 (2)	9.2 (12)	3.1 (4)	11.5 (15)
3rd quartile	26.5 (155)	27.1 (42)	25.8 (40)	18.1 (28)	5.8 (9)	5.2 (8)	0.6 (1)	17.4 (27)
4th quartile (highest)	29.7 (174)	20.7 (36)	23.6 (41)	24.7 (43)	9.2 (16)	2.9 (5)	2.9 (5)	16.1 (28)
**Job position** ^***^								
Head of the company, senior manager, or middle manager	9.6 (60)	30.0 (18)	10.0 (6)	31.7 (19)	11.7 (7)	5.0 (3)	1.7 (1)	10.0 (6)
Senior and intermediate level specialist	32.7 (205)	26.3 (54)	16.6 (34)	23.9 (49)	10.2 (21)	3.9 (8)	3.4 (7)	15.6 (32)
Service and sales worker	19.5 (122)	36.1 (44)	26.2 (32)	13.1 (16)	1.6 (2)	6.6 (8)	0.8 (1)	15.6 (19)
Skilled worker	27.3 (171)	20.5 (35)	41.5 (71)	12.3 (21)	2.9 (5)	8.8 (15)	1.8 (3)	12.3 (21)
Unskilled worker	11.0 (69)	31.9 (22)	26.4 (17)	5.8 (4)	2.9 (2)	14.2 (10)	5.8 (4)	14.5 (10)
**Work experience with current employer** ^*^								
<1 year	12.4 (77)	26.0 (20)	32.5 (25)	9.1 (7)	11.7 (9)	13.0 (10)	0.0 (0)	7.8 (6)
1 to 5 years	34.6 (214)	24.3 (52)	26.6 (57)	17.8 (38)	4.7 (10)	7.0 (15)	2.3 (5)	17.3 (37)
5 to 10 years	21.6 (134)	28.4 (38)	27.6 (37)	15.7 (21)	2.2 (3)	6.7 (9)	3.7 (5)	15.7 (21)
10 years and more	31.3 (194)	31.4 (61)	20.6 (40)	21.1 (41)	7.7 (15)	4.6 (9)	3.1 (6)	11.3 (22)

^*^p<*0.05*, ^**^p<*0.01*, ^***^p<*0.001*.

The analysis of the most frequent self-reported reasons for working while being ill shows significant differences in most sociodemographic groups. The gender of the respondents is the only factor with differences which are not statistically significant.

No possibilities for replacement have been reported more often by females than by males, and less frequently in the youngest age group. This reason is one of the most frequently reported ones in all education groups. Lack of replacement has been mentioned more often in lower-income groups (1st and 2nd quartile). Approximately one-third of heads of the companies, senior managers, middle managers, service and sales workers, as well as unskilled workers, recognized no possibility of replacement as the main reason for working while ill. Lack of the possibilities for replacement was slightly more often mentioned by respondents with longer work experience with the current employers if compared with groups having shorter experience (the highest number was observed among those whose work experience was 10 years and more).

Opposite to the lack of replacement, financial considerations have been mentioned more often by males than by females. Rather similar numbers were observed in the age groups, slightly lower numbers were identified in the youngest age group and age groups above 55 years. Financial considerations as the main reason were mentioned more often among respondents with lower educational levels, as well as in lower job positions, especially skilled workers, who mentioned financial considerations as the most frequent reason from all of the reasons mentioned in the study. Top occupation groups (head of the company, manager or specialist) consider financial considerations as the less frequent reason for presenteeism. Financial considerations were slightly more often reported by respondents with shorter work experience with the current employer (<1 year) if compared with the group with work experience is 10 years and more.

Specific aspects of work have been reported almost equally frequently in both genders and among all age groups but slightly more often in the age group 55–63 years. Along with the increase in the level of education, also the frequency of this reason for presenteeism increases. In general, different work-related aspects have been mentioned more often by higher income groups and among respondents with higher job positions—the frequency of this factor significantly decreases with each job position. Specific work aspects were less often mentioned by respondents with work experience <1 year with the current employers if compared with groups having 10 years and more experience.

Results on the sense of responsibility and feeling guilty were based on a small number of answers (28). This reason has been mentioned approximately equally by both genders. The only major difference was identified in the age group 64–80 years; this reason was mentioned as more frequent if compared to younger groups. This reason was more often reported by respondents with higher education levels and in the highest income quartile. Heads of the companies, senior managers, and middle managers, as well as senior and intermediate-level specialists, report a sense of responsibility and feeling guilty more often. This reason was considered important among respondents with <1-year of experience with their current employer.

Fear of losing a job, which was also a rather rare reason for working while being ill (analyses based on 44 answers), had been reported slightly more often by females than by males. This reason was a concern among the eldest groups and the youngest ones, with the highest proportion of the 64–80-year-olds reporting fear of losing a job. Respondents with higher education feel more confident in their workplace as only a few of them have reported fear of losing their job as the reason for working while ill. No other major differences were identified in other education groups. Fear of losing a job had been mentioned more often in lower-income groups and by unskilled and skilled workers. A slight tendency was observed in work experience—along with the increase in the work experience with the current employer, the number of respondents reporting fear of losing their job decreases.

Mild illness as the reason for working while being ill had been reported equally by both genders and almost equally by all education groups and job positions. Mild illness was most often used as an answer in the youngest age group. It has been mentioned less often in lower-income groups. The lowest percentage of respondents providing mild illness as the reason for working while ill was observed among those whose work experience was <1 year, but the highest—was among those whose experience was between 1 and 5 years.

The code “Did not want to visit the doctor” was used only for 16 answers, therefore, the results in different sociodemographic groups are based on very small numbers.

## 4 Discussion

The total prevalence of presenteeism found in our study (11.0%) is much lower than identified in another survey covering also data from Latvia (2). However, the results of both studies are not comparable due to methodological differences. Our studied population did not include self-employed persons, and previous research had already reported that the level of sickness presenteeism among the self-employed is higher than among workers who are employed by organizations (5, 29, 30).

In our study, a higher risk of sickness presenteeism was observed among females. This is consistent with other studies—women more often report both sickness presenteeism and sickness absence (2, 9, 18). Our results show that in both genders, three of the most frequent reasons were the same (no possibilities for replacement, financial considerations, and specific aspects of work), however, in females, no possibilities for replacement were the most frequent answer (29.4%), but, in males, financial considerations were reported most of them (30.0%). These findings are also consistent with previous evidence, women show greater concern for colleagues and worry about their workload (in our study this concern might be hidden under the answer “no possibilities for replacement”), meanwhile men—about money loss (31). Although other researchers have concluded that the differences in health-related behavior and gender roles can influence the level of presenteeism among genders differently, our results do not provide evidence to support such results. For example, delays in seeking help in case of illness have been mentioned by some researchers as one of the reasons among males (32, 33), but the results of our study show no gender differences for the answer “I did not want to visit a medical doctor” (2.3–2.7%). Among the reasons that influence the prevalence of sickness presenteeism in women noted higher participation in the annual health check, and readiness to seek advice from a medical practitioner (34), as well as work-family conflict is a stronger factor increasing presenteeism in females than males (31), which has been explained by the “double burden” (35).

The results of our study show that the odds of sickness presenteeism are higher for age groups 25–34 years (OR = 1.51) and 35–44 years (OR = 1.45) compared to the age group 18–24 years. Similar results have also been observed in other surveys; for example, the sixth EWCS report that older workers (aged 50 and over) are the least likely to report presenteeism (39%) if compared with workers under the age of 35 (44%) (2). This could be due to increased career responsibilities, financial pressures, and the need to maintain job security during this stage of life. Practically, this suggests that workplace interventions targeting this demographic should prioritize flexible work arrangements and financial support to reduce presenteeism behaviors. When looking for the reasons in age groups reported in our research, major differences were observed in the oldest and youngest group. For the oldest group (64–80 years), all three most frequent reasons are those which are not mentioned among the top three reasons in all other age groups—a sense of responsibility or feeling guilty and fear of losing a job were both mentioned by 27.3% of respondents and additional 18.2% stated they did not want to visit the doctor. Because this is the group of workers who have reached the retirement age in Latvia, there might be several reasons for such differences. The first thing is—good health is a precondition for staying active in the labor market after reaching retirement age (36). Thus, decreased presenteeism in this age group can be explained by the presence of a so-called healthy worker effect—after reaching retirement age, only those workers whose health is good enough continue working. In addition, one of the most important motives for working beyond retirement age is the financial benefit (36), therefore, fear of losing a job is one of the obvious reasons why workers of the retirement age work while being ill. Elder workers are often praised for good attendance, discipline, punctuality, loyalty toward the enterprise, reliable performance and commitment to quality (28, 37), and this can explain why the eldest group of respondents have given a sense of responsibility and feeling guilty as the reasons for working while ill.

The other group reporting differences in the reasons for working while ill was the youngest group. As the most frequent reason (25.6%) mild form of illness was mentioned. No possibilities for replacement were almost equally often reported by respondents aged 25–63 (27.0–30.3%). Some other studies have found that presenteeism is more common among young to middle-aged workers, presumably due to stronger attendance requirements for junior staff (15) or greater career-related concerns (3), so they would rather come to work than not. The codes used in our survey do not allow us to conclude anything related to the mentioned reasons. Likewise, we are also not able to provide any data regarding having children which has been identified as another major reason for presenteeism in middle-aged workers. Usually, workers having children prefer saving the use of sick leave (keep it for later) in case they need it in the future for child sickness or already have taken it often before (2, 35).

The results of our study show that senior and intermediate level specialists have higher unadjusted odds of presenteeism than skilled and unskilled workers (OR 0.76 and OR 0.80, respectively), but no significant difference remains after adjustment for gender, age, education, and survey year. A study on presenteeism in 35 European countries has shown similar results—presenteeism was higher in high job positions (2). One of the explanations is that managers and highly educated persons more often report that they are indispensable (21). The results of our study show that approximately one-third of heads of the companies, senior managers, middle managers, and service and sales workers (respondents belonging to both managerial groups) recognized no possibility for replacement as the main reason for working while being ill. A higher level of presenteeism among high job positions can be also explained by the fact that these workers aim to function as role models for their subordinates (14). Our results can support such explanation as the heads of the companies, senior managers, and middle managers as well as senior and intermediate level specialists have reported a sense of responsibility and feeling guilty more often (11.7 and 10.2%, respectively) if compared with groups of lower job position (1.6–2.9%). In addition, job responsibilities and engagement in different job positions play a significant role in the decision to work or not while being ill. Senior positions often have higher workloads and deadlines, which are frequently mentioned reasons to be sick at work (22). Data analysis in our research provide rather similar results—specific work-related aspects have been more important among respondents with higher job position; the frequency of this answer is almost six times less among unskilled workers if compared with top managers. Another finding should be highlighted—the fear of losing a job has been reported most often by unskilled workers (14.2%). This can be explained by job insecurity related to the perception of uncertainty about the job, a threat to current job status and problems in finding a new job that does not require specific skills (28).

However, other studies show no difference in presenteeism among white, pink, or blue-collar workers between social classes as managerial/professional, routine non-manual and skilled workers (5, 6). Some studies show the highest rate of sickness presenteeism in the office workers group (18). Those who work manual or physical work (assuming they have a lower education level, salary, or job position), usually have more physical health problems, for example, pain conditions caused by any reason (38). These health problems can simply deny the opportunity to do their job (thus reducing the possibility of sickness presenteeism), which cannot be said about those doing clerical/office work. Since our survey does not provide data on the specific type of illness that led to working while ill, nor does it allow us to assess its severity to determine whether respondents would have preferred sick leave over working while sick, this lack of data is one of our study's limitations. To sum it up, findings for different employment aspects as risk factors for sickness presenteeism are mixed, but there is evidence that the prevalence is greater in professional and highly skilled white-collar workers as taking time off to recover from minor illness is considered less legitimate in people with more senior roles in organizations (15), which is in line with our research data.

Sickness presenteeism odds tendencies in the education groups and salary quartiles are similar as these factors interrelate—presenteeism is higher in higher education and higher income quartiles. Significantly lower odds of presenteeism have only vocational secondary education (OR 0.74) compared to higher education, but no significant difference in other educational groups was found. Previous research in this area provides controversial results (6). Higher presenteeism in the higher education groups, which has been identified in several studies (7, 13) can be linked with less acceptance to take sick leave in senior positions or fewer replacement possibilities for higher positions (15). It has already been mentioned in the discussion that approximately one-third of heads of the companies, senior managers, middle managers, and service and sales workers (respondents belonging to both managerial groups) included in our analysis have recognized no possibilities for replacement as the main reason for working while being ill. Some other studies show a significantly lower risk of presenteeism in higher education resulting from higher health literacy (11) or no clear patterns in presenteeism about education (16). Low income increases the risk of sickness presenteeism because of loss of income due to absence and higher job insecurity, especially since workers are more prone to choose presenteeism in case of high unemployment when income or skill level is low (17). However, our findings regarding income do not fully support this as financial considerations as the main reason for working while ill have been mentioned rather equally by all income groups with a slightly higher frequency among the second income quartile (29.8%). Our results regarding education level partly support previous research as respondents with lower educational levels (except for the lowest education level based on a small number of respondents) more often report financial considerations than respondents with higher education levels. In addition, it is important to mention that our research has identified a tendency—along with the increase in the level of education, also the frequency of the specific work aspects as the reason for working while being ill increases. This can probably be linked to higher job positions, as discussed above.

The results of our analyses show that the risk of sickness presenteeism significantly increases with years worked with the current employer—the longer is work experience, the higher the risk of presenteeism. Other researchers have also identified the impact of work experience on sickness presenteeism—having a new job (recently changed company) is associated with less presenteeism, whereas presenteeism increases with each year of work experience which can be explained by increasing loyalty and identification with the employer (14) or increasing difficulties in the possibility of replacement (15). The data in our study do not allow us to assess the changes in the loyalty of respondents, but we identified that the lack of the possibilities for replacement was slightly more often mentioned by respondents with longer work experience with the current employers if compared with groups having a shorter experience which is consistent with the findings of other researchers. In addition, during the first year in a job, workers have to gain a reputation among colleagues and management to keep contracts permanent (20). Our results also support these findings as the differences in the answers regarding the self-reported reasons for working while ill were observed in our analysis. Respondents with <1 year of experience with their current employer reported a sense of responsibility and feeling guilty more often than other groups of respondents.

Presenteeism is a hazardous behavior that may have personal and organizational consequences, and future research on sickness presenteeism still faces many challenges. One of them is common sickness presenteeism metrics and presentee definition (number of episodes, duration of episodes, reasons, etc.) (23). The lack of such information was also one of the limitations we have identified in our study which does not allow us to provide a “dose-response” analysis and clear cross-national comparison. In addition, a 12-month recall period which was used in our study, is often used in many presenteeism studies (2, 5, 39). Similarly, to those studies, there might be recall bias also in our study. Another limitation of our study is the lack of information on the general health status of the respondents, the type of illness (e.g., infectious, musculoskeletal diseases or chronic conditions) during the sickness presenteeism episode and the severity of certain illnesses, which did not allow us to obtain results regarding adjustment for health status. Ill health is a significant predictor of sickness presenteeism, and researchers investigating presenteeism have identified a link between presenteeism and several health conditions such as allergies, hypertension, chronic pain, mental illness, migraines and arthritis (14, 40, 41). We also used coding of the answers to an open-ended question on self-reported reasons for working while ill. Although the same codes were used, the coding principles were the same; the supervision of the coding process in all surveys was done by the same researcher who had been involved in the coding of the answers from the first survey, and the persons who coded were different and that might have influenced the results, however future research could benefit from qualitative methods to explore underlying motivations for presenteeism more comprehensively. Furthermore, a cross-sectional study is commonly considered as a limitation for analysis of sickness presenteeism. To overcome this limitation, a sampling method to represent the entire working population of Latvia was used.

Despite some researchers have predicted that the prevalence of sickness presenteeism will remain constant or might even increase with regard to the future workforce (12), we would like to stress that the COVID-19 pandemic has changed work patterns, including growing telework use and how organizations and individuals changed their concept of work while ill, will also leave a mark on presenteeism (42, 43). Sickness presenteeism changed significantly during the COVID-19 pandemic (44) due to heightened health awareness, remote work, changes in mental health and strict policies around illness. Sickness presenteeism perception is transformed in the telework context, and most probably increase workers' likelihood of working while sick. During the pandemics organizations enforced stricter health and safety rules, preventing employees from coming to work sick, as well as employees became more conscious of the risks of spreading illness, leading to a cultural shift where staying home when sick became more acceptable. However, many employees worked from home while sick, making presenteeism less visible but still prevalent (45, 46). Remote work also blurred the lines between rest and work, making presenteeism harder to track (47). It is indicated that lack of physical presence at the premises of the employer makes it harder to justify the need for formal sick leave, so workers might choose to continue working despite feeling unwell (42). However, for the workers whose work content does not allow telework, presenteeism might decrease because strict epidemiologic requirements have been introduced in many countries, including a stay-at-home policy when you face symptoms related to the possible COVID-19 infection. Therefore, the data on presenteeism of the next (fifth and sixth) national surveys *Work Conditions and Risks in Latvia* should be analyzed in the context of changes in presenteeism over the whole period.

## 5 Conclusions

The results of our studies highlight significant differences in prevalence and self-reported reasons for sickness presenteeism in most demographic groups. Therefore, to reduce sickness presenteeism in companies, employers need to analyze the structure of the workforce in their companies, accordingly revise sickness absence management and use different communication messages to encourage workers to stay at home while being ill. We consider that future studies are needed to extend research on reasons for sickness presenteeism in other countries and different types of working environments. Research should also be conducted to evaluate the effects of the COVID-19 pandemic on changes in prevalence and reasons for sickness presenteeism.

## Data Availability

Publicly available datasets were analyzed in this study. This data can be found at the following original datasets from the three consecutive surveys Work Conditions and Risks in Latvia were are available here: (1) from 2006—https://doi.org/10.48510/FK2/EYHQZ0, (2) from 2010—https://doi.org/10.48510/FK2/1JA7AR, and (3) from 2013—https://doi.org/10.48510/FK2/6ZRDUY. The original dataset from the study Work Conditions and Risks in Latvia from 2018 is available upon an official data request from the State Labor Inspectorate, which is the owner of the data (https://www.vdi.gov.lv/en).
